# Pretreatment-Integration for Milk Protein Removal and Device-Facilitated Immunochromatographic Assay for 17 Items

**DOI:** 10.1038/s41598-019-47692-6

**Published:** 2019-08-12

**Authors:** Zhiwei Qie, Ziwei Huang, Zichen Gao, Wu Meng, Yanhui Zhu, Rui Xiao, Shengqi Wang

**Affiliations:** Beijing Institute of Radiation Medicine, Beijing, 100850 People’s Republic of China

**Keywords:** Assay systems, Biosensors

## Abstract

Accurate and comprehensive immunochromatographic assay (ICA) data are urgently required in the daily supervision of plants, schools, testing institutions, and law-enforcing departments. Through pretreatment-integration and device-facilitated operation, a quantitative ICA with high sensitivity and throughput was realized on the basis of a commercialized semi-quantitative ICA strip. Three pretreatment methods, namely, acid base, heavy metal salt, and organic solvent methods, have less than three steps. The pretreatment was established for protein removal. A total of 17 pretreated ICA items in milk were considered for the identification of the most suitable pretreatment method. The items are composed of six items pretreated by the acid-base method, six by the heavy salt method, and five by the organic solvent method. Then, the ICA results with pretreatment were compared with those without pretreatment. After pretreatment, the signal intensity increased by 39%, the detection limit decreased to 12%, the half maximal inhibitory concentration decreased to 18%, and the detection range increased fourfold. A device with mixing and centrifugation functions was designed for the pretreatment-related operations. A pre-incubation sampling device was used to facilitate incubation in batch and high-throughput detection. An ICA reader was used. The detection throughput reached 8 samples per batch or 32 samples per hour. The designed devices were printed through 3D printing and rapid prototyping.

## Introduction

Based on the principle of specific antigen-antibody recognition, immunoassay is a powerful method for screening samples^1^. In contrast to chromatography-based methods, immunoassays are simple, specific, and easy to use, and do not require costly instrument^2,3^. Thus, these assays are suitable to on-site detection. Immunochromatographic assay (ICA) technology, a combination of chromatography and immunochemical reactions, has attracted attention from researchers^4,5^. This technology does not require additional precipitation or washing steps because of the separation of reacted products from unreacted products through fluid movement in the strip (e.g., sorbent and membrane). ICA has been used in detecting hormones, drugs, and pesticide toxics in various fields, such as food safety and quality control^6–12^. However, it has relatively low sensitivity (typically reporting semiquantitative results) and is capable of only single-target detection and vulnerable to the matrix effect. Hence, a rapid and applicable pretreatment is necessary for high-throughput detection. The inconsistency of pretreatment methods in milk ICA, has become a restrictive factor for high-throughput detection. In this study, 17 ICA items in milk were selected according to manual instruction. Of these items, two must be diluted five times by PBS buffer before ICA detection, ten must be pre-incubated for two minutes at room temperature, and five must be detected directly and even required extraction (Table 1).

**Table 1:** Comparison of ICA results with or without proposed pretreatment of 17selected items in milk.

Item	Pretreatment in this work	Pretreatment in commercial ICA kit	Ratio of IC_10_^a^	Ratio of IC_50_^a^	Ratio of IC_80_/IC_20_^a^	Pretreatment in reference
Zearanol	Acid-base method	PBS Dilution, 5 times	0.23	0.27	1.14	Extraction^c^ ^37^
Trimethoprim	Acid-base method	Preincubation 2 min, RT^b^	0.13	0.45	4.11	PBS Dilution, 10 times^38^
Danofloxacin	Acid-base method	No pretreatment	0.32	0.40	1.08	Centrifugation, 48 °C^39^
Aflatoxin M_1_	Acid-base method	PBS Dilution, 5 times	0.26	0.28	0.99	PBS Dilution, 5 times^40^
Florfenicol	Acid-base method	Preincubation 2 min, RT	0.81	1.45	1.86	PBST Dilution, 4 times^41^
Melamine	Acid-base method	No pretreatment	0.92	1.45	2.01	Extraction^42^
Flunixin meglumine	Organic solvent Method	Preincubation 2 min, RT	0.31	0.70	3.13	Extraction^43^
Benzylpenicillin potassium	Organic solvent Method	Preincubation 2 min, RT	0.12	0.18	1.68	Extraction^41^
Tetracycline	Organic solvent Method	Preincubation 2 min, RT	0.53	0.53	0.98	HEPES Dilution, 20 times^44^
Spectinomycin	Organic solvent Method	No pretreatment	0.86	0.99	1.35	NR^d^
Sulfadimidine	Organic solvent Method	No pretreatment	0.09	0.19	2.53	PBS Dilution, 5 times^45^
Lincomycin	Heavy metal salt Method	Preincubation 2 min, RT	0.80	0.97	0.81	PBS Dilution, 4 times^46^
Erythromycin	Heavy metal salt Method	Preincubation 2 min, RT	0.80	0.90	1.14	PBS Dilution, 3 times^47^
Chloramphenicol	Heavy metal salt Method	Preincubation 2 min, RT	0.92	0.77	1.35	Centrifugation and Dilution, 20 times^48^
Thiamphenicol	Heavy metal salt Method	Preincubation 2 min, RT	0.99	1.14	1.24	PBST Dilution, 4 times^46^
Kanamycin	Heavy metal salt Method	No pretreatment	0.50	0.46	1.01	Centrifugation and Dilution, 2 times^49^
Dexamethasone	Heavy metal salt Method	Preincubation 2 min, RT	0.82	0.86	1.11	Centrifugation and 25% methanol^50^

^a^Ratio of IC value of with proposed pretreatment divided by that pretreatment in commercial ICA kit; ^b^Room tempeture; ^c^Method based on non-ICA immunoassay; ^d^Not reported.

Milk is an important source of high-quality proteins for humans. It is the core material of yogurt, cheese, and other products. Milk contaminants affects the milk quality and pose a threat to human health. Potential chemical contaminants in milk include veterinary drugs, hormones, mycotoxins, and adulterants^13^. The European Union and China have determined the maximum limit of chemical pollutants in dairy products^14,15^ to ensure the quality of dairy products and prevent the adverse effects of these pollutants. Many methods for detecting chemical contaminants in dairy products have been established^16,17^.

ICA has become the subject of increasing research attention^18^. Milk samples are traditionally analyzed directly or after pretreatments, such as dilution, protein precipitation, and centrifugation^19,20^. Satisfactory recoveries (86–106%) have been obtained^8^. However, dilution decreases the target concentration and increases the chance of false-negative values. Protein precipitation, usually realized through the use of strong acids, such as trichloroacetic acid, organic solvents (e.g., methanol, acetonitrile, and dichloromethane), or heavy metal salts (e.g., lead acetate), is typically accompanied by a time-consuming liquid–liquid or solid-phase extraction to increase detection sensitivity^21–31^. Protein removal by centrifugation relies on a high speed, which is difficult to attain on site. Simple sample pretreatment procedures are vital to successful rapid testing but are frequently overlooked by researchers developing assays. No systematic investigation on milk pretreatment against the existing ICA strip has been reported.

In this work, three pretreatment methods, namely, acid-base, heavy metal salt, and organic solvent methods, for ICA detection in milk were established. The methods were mainly of practical innovation. Compared with a lengthy and complex pretreatment in traditional confirmatory detection, the modified pretreatment methods contained less than three steps, namely, protein denaturalization, neutralization, and centrifugation, which take less than eight minutes and require no further extraction. Furthermore, a device with mixing and centrifugation functions was used for pretreatment. The obtained supernatants were directly used for ICA, and the results with and without pretreatments were compared on the basis of limit of detection (LOD), the relative signal intensity of the reflected light, and detection range. A total of 17 pretreated ICA items in milk were considered for the identification of the most suitable pretreatment method. The items are composed of six items pretreated by the acid-base method, six by the heavy salt method, and five by the organic solvent method. A pre-incubation sampling device that incorporates synchronous pre-incubation with sampling was designed to eliminate the difference in pre-incubation procedure and facilitate a high-throughput detection with ICA reader. The designed devices were printed by 3D printing rapid prototyping.

## Methods

### Chemicals

Standard chemicals, namely, danofloxacin, erythromycin, sulfadimidine, benzylpenicillin potassium, chloramphenicol, lincomycin, tetracycline, spectinomycin, thiamphenicol, dexamethasone, kanamycin, melamine, trimethoprim, zearanol, aflatoxin M_1_, flunixin meglumine, and florfenicol, were purchased from Sigma (St. Louis, MO, USA). All the chemicals used were of analytical grade. Lead acetate, potassium oxalate, disodium hydrogen phosphate, trichloroacetic acid, sodium hydroxide, and dichloromethane were obtained from Sinopharm Chemical Reagent Co., Ltd. (China). High-purity water was obtained from a Milli-Q water system (Millipore, Billerica, MA, USA). Pure milk was purchased from a local supermarket. Commercialized semiquantitative ICA strip and an ICA strip reader were supplied by Beijing Meizheng Bio-Tech Co., Ltd. (Beijing, China)

### Solution preparation

Lead acetate solution (20%, w/v) was prepared by dissolving 20 g of lead acetate in 100 mL distilled water. A potassium oxalate buffer solution was prepared by dissolving 3 g of potassium oxalate and 7 g of disodium hydrogen phosphate in 100 ml distilled water. Sodium hydroxide (5 mol L^−1^) was prepared by dissolving 4 g of sodium hydroxide in 100 mL of distilled water. All the solutions were prepared and stored at room temperature until use.

### Pretreatment methods

#### Acid-base method

Exactly 10 mg of trichloroacetic acid was added to 1 mL milk for protein precipitation. After denaturation (3 min) under vibrate oscillation (SCLOGEX, USA) and centrifugation (5000 rpm, 3 min; Centrifuge 5424, USA), the pH of the obtained supernatant was neutralized with NaOH (5 mol L^−1^, 2.5 μL per 100 μL supernatent), centrifuged (5000 rpm, 3 min), and subjected to ICA (100 μL per assay).

#### Organic solvent method

Dichloromethane of equal volume was used in milk protein denaturation. The mixed solution was thoroughly oscillated for 3 min. The supernatant obtained after centrifugation (5000 rpm, 3 min) was directly used in the ICA (100 μL per assay).

#### Heavy metal salt method

A lead acetate and potassium oxalate buffer system has been reported and modified^32^. Briefly, the lead acetate solution of 150 μL was added to 1 mL of the milk sample for protein denaturation. After fully mixing for 2 min, 100 μL of potassium oxalate was added to neutralize the unreacted lead acetate. Precipitation and neutralization were completed in 3 min. The supernatant obtained after centrifugation (5000 rpm, 3 min) was used in the ICA.

#### Screening of the pretreatment and data analyses

The ICA result was a rebalance of chromatographic conditions, properties of colloidal gold, and pre-immobilized antigens on strip. According to the manual of the commercialized ICA kit, ICA result was obtained 6–7 min after sampling. After pretreatment, the sample viscosity was reduced for protein removal, and the ICA system was rebalanced. Thus, the readout time interval was re-determined. The ICA data of the spiked samples with and without pretreatments were compared, and a proper pretreatment with high sensitivity, high intensity, and wide detection range was selected.

According to the cut-off value([C]) of the commercialized ICA strips, the analyte was serially diluted for the acquisition of the final working standard solutions (8*[C], 4*[C], 2*[C], [C], 0.5*[C], 0.25*[C], 0.125*[C], 0 ppb). The spiked samples were subjected to different pretreatment methods, and the control group was treated in accordance with the guidelines of the commercialized ICA kit. The samples with or without pretreatment were incubated with microhole-packed colloidal gold labeling materials according to the guidelines of the commercialized ICA kits before sampling. The assay was conducted in triplicate, and the results were compared. The experimental signals were normalized by using Equation (1).

1 $${\rm{Normalized}}\,{\rm{response}}=\frac{B-{B}_{\infty }}{{B}_{0}-{B}_{\infty }},$$

where *B* is the signal determined in the increasing concentration of an analyte, *B*_*∞*_ is the inhibited signal under an excess concentration of analyte, and *B*_0_ is the negative signal with no analyte spiked. The normalized response was plotted as a function of the analyte concentration, and the experimental data of the calibration curve were fitted to Equation (2).

2 $${\rm{Inhibition}}={A}_{2}+\frac{{A}_{1}-{A}_{2}}{1+{(\frac{X}{{X}_{0}})}^{\Lambda P}}.$$

The LOD was defined as the 10% maximum inhibition concentration^33,34^. The LOD (IC_10_), IC_50_, and the detection range (IC_20_–IC_80_) were calculated in accordance with the regression equation. Optimal pretreatment methods were screened for the 17 selected commercialized ICA kits against the potential chemical pollutants in milk on Chinese market.

#### Auxiliary apparatus

**Figure 1:**
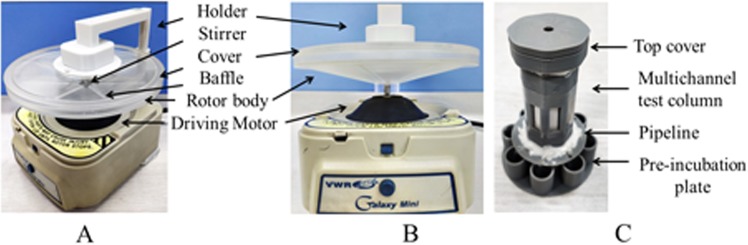
Mixing–centrifugation device (**A**,**B**). Pre-incubation sampling device (**C**).

**Figure 2:**
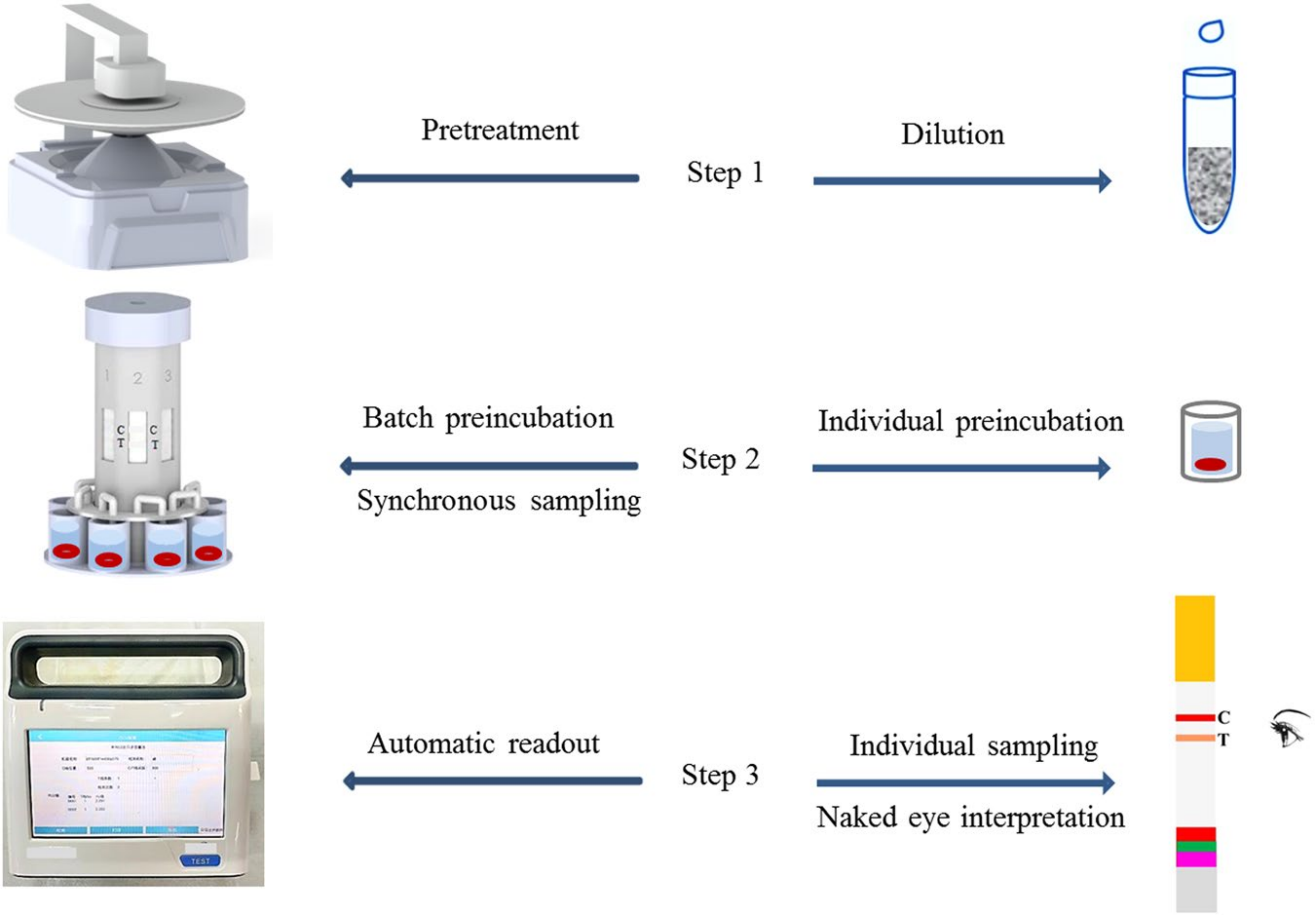
Comparison of the ICA process in this work (left) and the traditional approach (right). Step 1 shows the pretreatment of the milk sample. Three pretreatment methods were investigated and performed through the designed mixing–centrifugation device and compared with the traditional way of simple dilution. Step 2 demonstrates the batch pre-incubation in comparison with the traditional individual pre-incubation. Step 3 shows the high-throughput detection through synchronous sampling and automatic readout. By contrast, traditional detection includes individual sampling and naked eye interpretation.

The ICA results were readout by a strip reader. Pretreatment efficiency was improved by modifying the mixing–centrifugation device (Fig. 1A,B) so that it can perform all the pretreatment operations. The modified device was made from a portable centrifuge in our laboratory. Then, a pre-incubation sampling device (Fig. 1C) that incorporates synchronous pre-incubation with synchronous sampling was designed. The mixing–centrifugation device contained three parts: a holder, rotor (the upper part is a compound stirrer, the middle is the top cover of the rotor body, and the lower part is the baffle-attached rotor body), and driving motor. The pre-incubation sampling device is mainly composed of a top cover, pre-incubation plate, and batch of pipelines, which form an airtight system with a multichannel test column. The working process of the three devices is introduced in the Results section. The comparison between the ICA process in this study and the traditional approach is illustrated in Fig. 2. Device prototypes were created by using a stereo lithography-type 3D printer (HORI, China). Designs were produced as.sldasm and printed with a UP Fila photopolymer.

## Results

### Acid-base method

**Figure 3:**
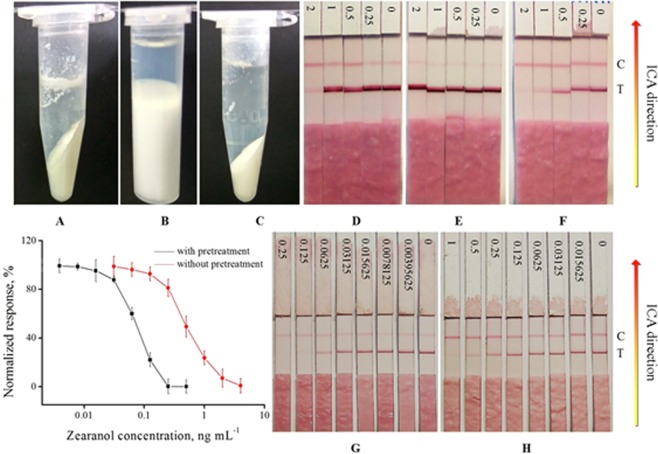
Supernatant (upper layer) of spiked milk samples obtained after pretreatment: acid-base method (**A**), organic solvent method (**B**), heavy metal salt method (**C**). ICA result of zearanol spiked milk with acid-base method (**D**,**G**), heavy metal salt method (**E**), and without pretreatment (**F**,**H**). The spiked concentrations were 2, 1, 0.5, 0.25, and 0 μg L^−1^ for (**D**–**F**); 0.25, 0.125, 0.0625, 0.03125, 0.015625, 0.0078125, 0.00395625, and 0 μg L^−1^ for (**G**); and 1, 0.5, 0.25, 0.125, 0.0625, 0.03125, 0.015625, and 0 μg L^−1^ for (**H**) (left to right). The C and T on the right side represent the control and test lines, respectively. The calibration for zearanol spiked milk with and without pretreatments is shown in the lower left. The strips for zearanol in (**D**–**H**) were form different supplier.

Different amounts of trichloroacetic acid were added to the milk samples and compared. The results showed that 10 mg is the minimum amount to get clear supernatant (data not shown). Approximately 700 μL of supernatant was obtained per milliliter of pure milk (Fig. 3A). The acid-base method involved three sequential steps, namely, protein denaturalization, neutralization, and centrifugation, which were completed in less than 9 min. The protein denaturation and two times of centrifugation were realized on the mixing–centrifugation device under different speeds. The absence of the need for traditional complex manual operation obviously saved operation time. In this method, no dilution effect was induced by the reagent because trichloroacetic acid was solid, and 2.5 μL of NaOH per 100 μL of supernatant is negligible. The acid-base method was applied at the following conditions: 1. the target was acid-resistant, 2. the milk protein could be precipitated effectively, and 3. the supernatant could be directly used for ICA detection after neutralization and centrifugation. Among the the 17 selected chemical pollutants, six items, namely, mycotoxin aflatoxin M_1_, additive agent melamine, hormone zearanol, veterinary drug trimethoprim, danofloxacin, and florfenicol (Table 1) were fit for the acid-base method. Aflatoxin M_1_ (AFM_1_) is a class I carcinogen and the most monitored item in milk. Melamine is also routinely monitored, especially after the death of six infants and many cases of kidney stones in Chinese infants and children that ingested milk adulterated with melamine in September 2008. Hormone and veterinary drugs could cause considerable health damage, and three drugs, namely, trimethoprim, danofloxacin, and florfenicol, are the typical pollutants of the sulfanilamide group, quinolones, and chloramphenicol, respectively. Therefore, a wide range of milk pollutants could be covered by ICA detection with the acid-base pretreatment.

### Organic solvent method

Dichloromethane was used as a denaturant but not an extract reagent. The amount of dichloromethane was optimized and the equal volume is the minimum dosage. Approximately 300 μL of supernatant per milliliter of pure milk could be obtained (Fig. 3B). The pretreatment involved two steps, namely, protein denaturalization and centrifugation, which could be completed in less than 5 min. No dilution effect was induced by the reagent, and the organic solvent method was suitable for the following conditions: 1. the target was insoluble in organic solvent, 2. the organic solvent was incompatible with water, 3. the milk protein could be denaturalized effectively, and 4. the supernatant could be directly used for ICA detection after centrifugation. Five items got better ICA results with the organic solvent method as pretreatment. The five items covered four groups of antibiotics and one antiphlogistic drug, indicating that the organic solvent method helps in pollutant screening in veterinary drugs (Table 1).

### Heavy metal salt method

The dosage of lead acetate and potassium oxalate buffer was optimized and the selected amount was sufficient for denaturalization. No coagulation was induced. Approximately 650 μL of the supernatant could be obtained per milliliter of pure milk (Fig. 3C). This pretreatment included three steps that could be completed in 6 min. The heavy metal salt method was favorable for the following conditions: 1. the target did not react with heavy metal salt, 2. the milk protein could be precipitated effectively, and 3. the supernatant could be directly used for ICA detection after centrifugation. Six items were fit for the heavy metal salt method. The six items contained five antibiotics and one hormone, and the antibiotics were different from those in the organic solvent method. These results suggested that the pollutant could be screened in different groups of veterinary drugs and hormones after the heavy metal salt method (Table 1). The addition of 250 μL of reagent to 1 mL of spiked milk induced a certain dilution effect but better ICA results were obtained. These results proved the effectiveness of the method.

### Pretreatment screening and data analysis

**Figure 4:**
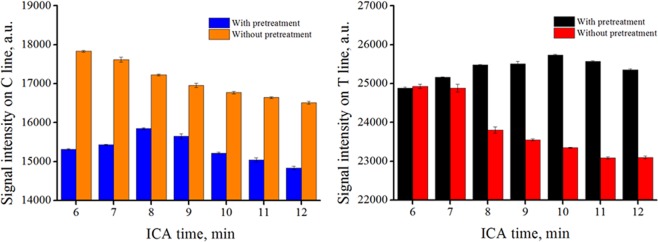
T and C line values of samples with and without pretreatments. The commercialized ICA results were readout in 6–7 min. In the bar chart, the T and C line values without pretreatment decreased obviously after 8 min. By contrast, for the sample with pretreatment, the T line value remained relatively stable in 8–12 min, whereas the C line value remained relatively stable in 6–10 min. A high T line value and a low C line value were obtained after pretreatment for zearanol spiked samples.

The ICA result with pretreatment was a rebalance of the traditional ICA process. Thus, we focused mainly on the change in the ICA result. After pretreatment, the viscosity of the sample was reduced, and chromatographic speed increased. The time interval of the ICA readout was re-determined. In Fig. 4, for samples without pretreatment, the test line (T line) and control line (C line) values remained relatively stable in 6–7 min and decreased sharply after 8 min. By contrast, the T line values remained relatively stable in 8–12 min for the samples with pretreatment, whereas the C line value remained relatively stable for 6–10 min.

Thus, the readout time interval for the sample with pretreatment was selected as 8–10 min. In this study, all the values were collected at 8 min after sampling and analyzed. Milk showed obvious matrix effect that interfered with the gold-labeled material combined on the test and control lines. The samples with pretreatment had high T and low C line intensities, implying that the colloidal gold and antibodies pre-immobilized on the commercial ICA can be recognized more effectively. The amount of gold-labeled material that combined on the test line increased, whereas the amount of material combined by the control line decreased. Thus, different color stability windows were observed. This result suggested that the possible negative effect, like interference with immune recognition, of the chemical reagents used in the pretreatment method was negligible.

The traditional ICA strip contains four parts. Figure 3D depicts the sample pad, conjugate pad, nitrocellulose membrane, and absorbent pad (from bottom to top). The sample and conjugate pads overlapped and were located at the lower part of the entire strip. The nitrocellulose membrane was approximately at the middle and contained the T and C lines. The absorbent pad located at the top was marked with spiked concentration. From left to right, Fig. 3D demonstrates five ICA strips with decreasing spiked concentrations, which produced stronger T line and weaker C line. Pretreatment screening was performed by first analyzing the target property and comparing the ICA results of different pretreatments. For example, tetracycline is not acid resistant and can react with heavy metal ions to form precipitation. Thus, the ICA data obtained by the traditional approach and organic solvent method were compared. Lincomycin is soluble in water, acid and base resistant, and insoluble in dichloromethane. Thus, the three pretreatments and traditional assay were conducted and compared. Zearanol is soluble in dichloromethane and thus unfit for the organic solvent method because dichloromethane is intended to act as a denaturant but not an extract reagent. Thus, the pretreatment method was only compared with the acid-base, heavy metal salt, and traditional ICA methods. Figure 3 exhibits that, in comparison with the commercial ICA method for zearanol (Fig. 3F,H), the T line intensity was significantly enhanced (Fig. 3E) after lead acetate pretreatment, but no obvious inhibition was observed, suggesting that the ICA sensitivity decreased significantly. However, after acid-base pretreatment, the inhibition trend could be clearly observed (Fig. 3D,G). The signal intensities of the test lines were significantly different between D and G because the strips for zearanol in D and G were form different suppliers. Difference (line intensity and target-induced inhibition) in sensitivity among D and F and G and H, and higher ICA was observed between G and H after pretreatment. The calibration data based on the acid-base method and commercialized test kit were further compared (Fig. 3). The nonlinear fitting curve of the former was y = 98.9974–99.5939/(1 + (x/0.0698)^1.8872^). The IC_50_, corresponding to 50% inhibition of the reflection intensity of the negative control was 0.0710 ng mL^−1^. LOD, defined as 10% inhibition of that of the negative control was 0.0266 ng mL^−1^, and the detection range (IC_20_–IC_80_) was 0.0342–0.1501 ng mL^−1^. R^2^ was 0.9966, and the nonlinear fitting curve of the latter was y = 98.0912 −100.3804/(1 + (x/0.2566)^2.0491^). The IC_50_ was 0.2673 ng mL^−1^, LOD (IC_10_) was 0.0981 ng mL^−1^, the detection range (IC_20_–IC_80_) was 0.1392–0.8416 ng mL^−1^, and R^2^ was 0.9964. Compared with the latter, the IC_50_ of the spiked samples with pretreatment decreased to 27%, the LOD decreased to 23%, the detection range was 1.14 times wider (IC_80_/IC_20_ without pretreatment divided by that with pretreatment), and R^2^ improved.

The abovementioned analysis indicates that the acid-base method is the optimal pretreatment for zearanol spiked samples. Similarly, the proper pretreatment for the selected 17 ICA items against milk on the Chinese market was screened, analyzed, and summarized in Table 1. The calibration values for the other 16 items are provided in the Supplementary Information.

In Table 1, the ICA results with proposed pretreatment were essentially improved. For example, the LOD (IC_10_) of benzylpenicillin potassium could decrease to 0.12, and its IC_50_ value to 0.18. The detection range of trimethoprim could be widened 4.11 times, but the IC_50_ values of florfenicol, melamine, and thiamphenicol were slightly increased.

In general, increase in flow speed could result in low color intensities due to the decreased efficiency in capturing labeled antibodies at the test- and control-lines. But the high lateral flow speed in this work was accompanied by the decreased matrix effect produced by the three pretreatment methods. Advantageous ICA results validated the necessity and efficiency of the pretreatment. Interestingly, we also observed that the ICA results and the optimal pretreatment varied with ICA strip source like the ICA result between D and F and that between G and H in Fig. 3, thereby the improved ICA results are inseparable from the screening of proper ICA strips. This finding suggested that the ICA result is a balance among all the factors, including the pretreatment methods, physical and chemical properties of the item, and inherent characteristics of the ICA strip itself.

For the blind sample detection, a proper pretreatment method should be selected on the basis of the items to be screened. Furthermore, the detection range may not cover its concentration. A proper dilution ratio should be selected through serial dilution until a 50% inhibition of ICA is observed. Then, the concentration could be calculated through the calibration curve.

For the sample of trace target concentration, condensation does not usually occur because a concentration lower than the detection range is considered acceptable. The proposed pretreatment methods integrated various pretreatment methods into merely three, thus paving the way for the high-throughput detection.

### Auxiliary apparatus

**Figure 5:**
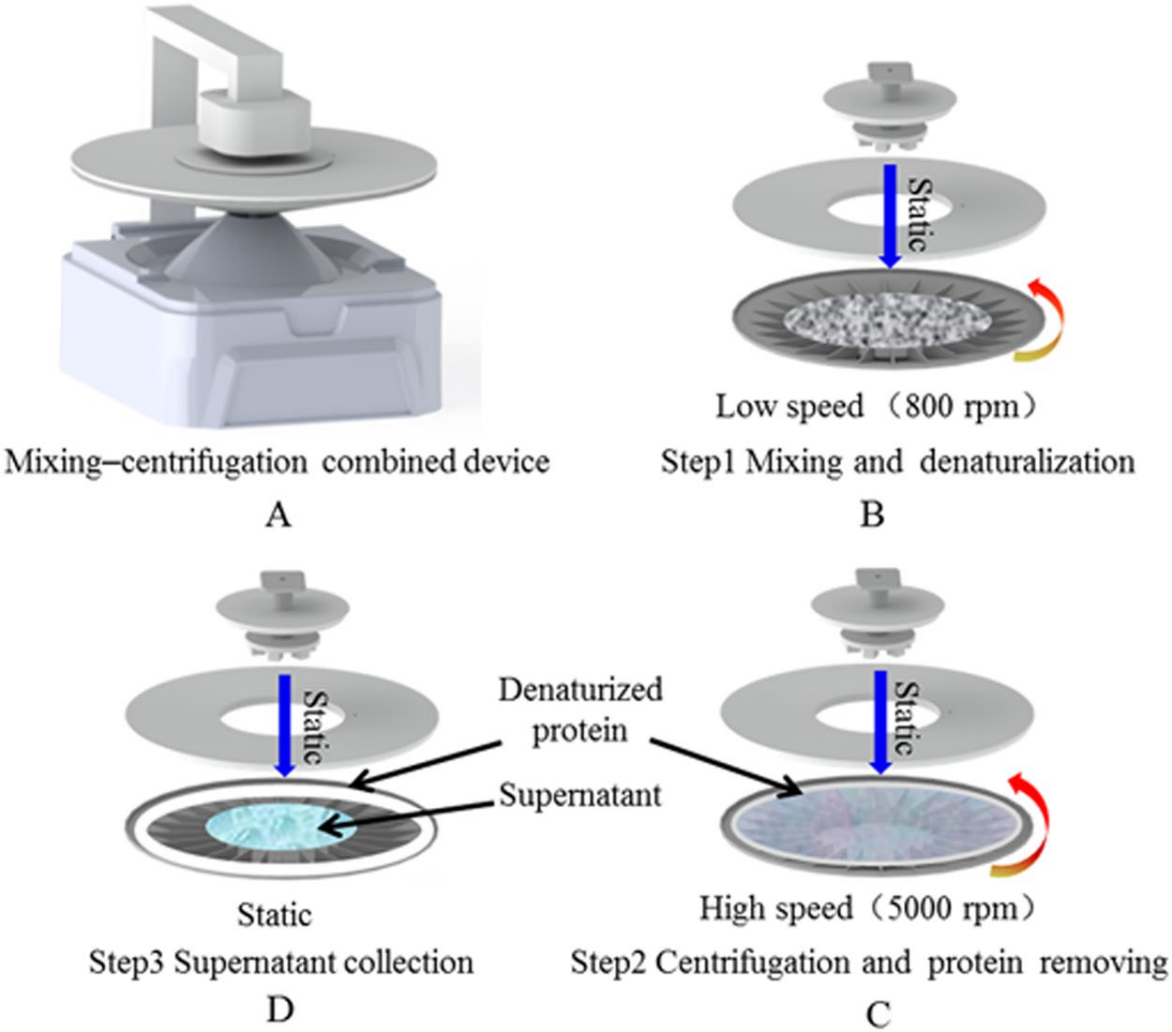
Schematic diagram for mixing–centrifugation device (**A**) and the working process. At low speed (**B**, Step 1), the milk sample was mixed with a chemical reagent, and the protein was denaturalized. At high speed (**C**, Step 2), the denaturalized protein was thrown away to the edge of the rotor body by centrifugal force. When the rotor stopped (**D**, Step 3), the supernatant aggregated to the center of the inverted-shaped rotor body by gravitational force. To aid rapid prototyping and redesign, 3D printing was used.

The mixing–centrifugation device could conduct all the pretreatment operations, mixing, denaturalization, and centrifugation (Fig. 5A). Briefly, mixing and centrifugation were realized through the relative motion of the static stirrer and the rotor body at different rotational speeds. At low speed (Fig. 5B), the milk sample fully mixed with a chemical reagent, and the protein was denaturalized. At high speed (Fig. 5C), the denaturalized protein was scattered to the edge of the rotor body through centrifugal force. The inverted conical rotor facilitated supernatant aggregation after centrifugation through gravity (Fig. 5D). The baffle structure arranged at the rotor bottom was intended to assist the acceleration of liquid.

**Figure 6:**
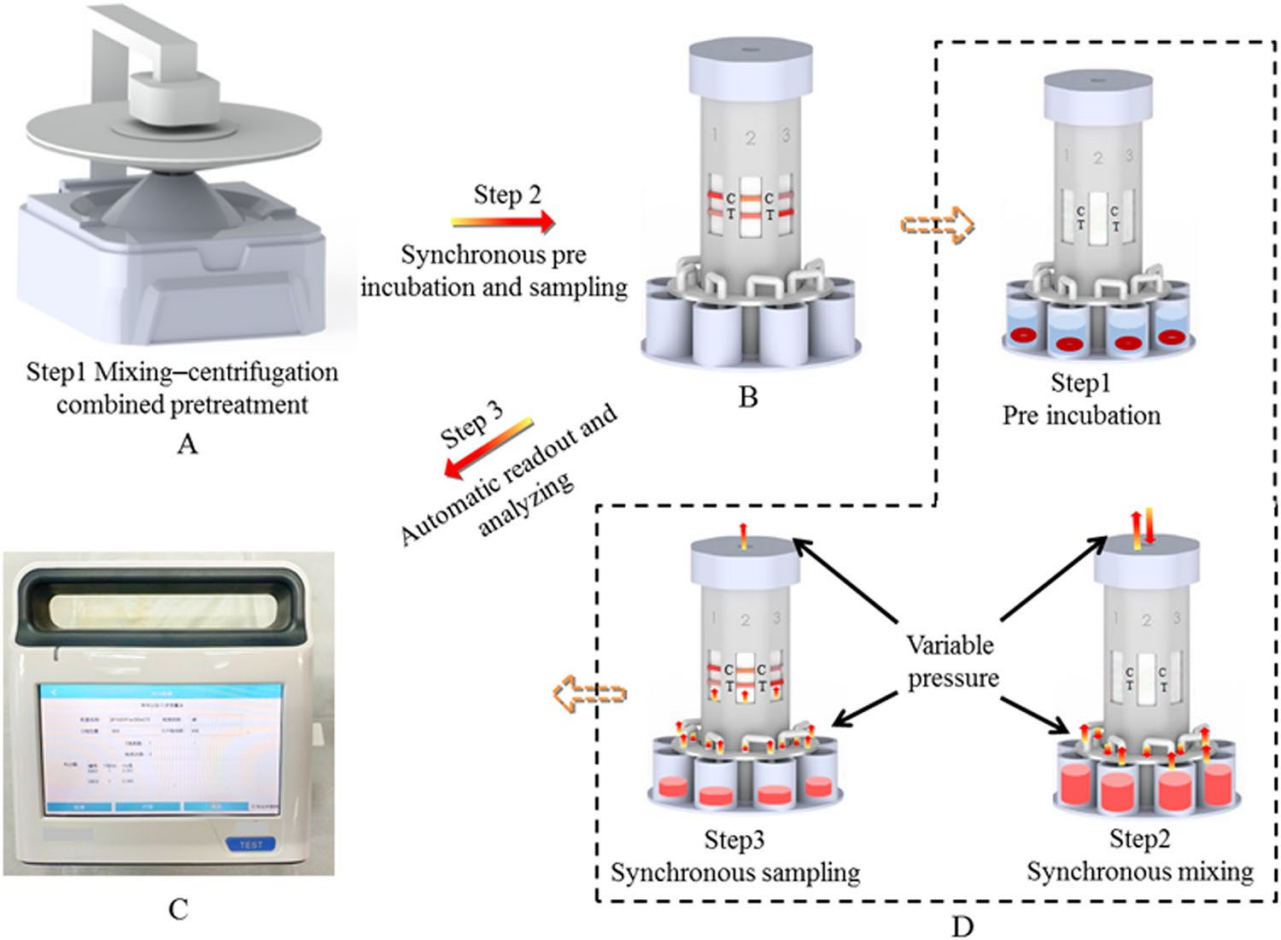
Process of pretreatment-integration and device-facilitated operation Pretreatment was carried out using mixing-centrifugation device (**A**, Step 1), followed by synchronous pre incubation and sampling (**B**, Step 2). Pre incubation also include 3 steps, pre incubation, synchronous mixing and sampling by sequence (**D**). The results were read out and analyzed by a designed ICA reader (**C**, Step 3).

The strip reader readout the ICA signal and the four-parameter logistic curves were fitted to the data according to the method outlined by Holstein^35,36^. The eight-channel test column was composed of a plurality of channels that contain ICA strips. A pre-incubation sampling device was designed for synchronous sampling, and the device is shown in the lower part of the Fig. 6B. The mixture in the incubation hole could be synchronously mixed and sampled by the variable pressure provided from the top of the test column and applied to the sampling plate through the pipeline (Fig. 6D).

As previously mentioned, the ICA results remained relatively stable in 8–10 minutes, which was sufficient for readouts (typically eight ICA strip per batch taking 10 seconds to readout). Thus, the swift sequential readout would have negligible effect on the ICA results.

Combining the pretreatment together, the detection throughput could reach 32 samples per hour, in which the pretreatment consume no more than nine minutes on average per batch. By contrast, the traditional ICA takes at least six minutes per assay, and the detection throughput would not exceed 10 samples per hour.

Through the established pretreatments and designed devices in this study, the ICA detection throughput for chemical contaminants in dairy was no longer limited by the inconsistent pretreatment methods, and the time error caused by manual operation.

## Discussion

The methods proposed in this work were beneficial to practical use. Pretreatment integration helps in realizing an ICA with high sensitivity and quantitative detection by using existing commercialized semiquantitative ICA strips. Supplementary devices further improve the throughput of pretreatment and detection. After pretreatment, the sensitivity was improved fivefold, and eight ICA results could be readout in sequence in one batch, with the detection throughput improved from less than 10 to a minimum of 32 per hour, taking pretreatment together. The improved sensitivity was crucial, especially for the trace detection of prohibited items and the accurate interpretation for items with maximum residue limit (MRL) by authorities. To the best of our knowledge, this study is the first work focusing on the pretreatment-integration and device-facilitated operation of milk ICA. More accurate and comprehensive ICA data based on existing commercialized ICA strips would be helpful, especially for units, such as plants, schools, testing institutions, and law-enforcing departments. Additional work should be done for the development of a smart and fully automatic food-safety monitoring platform that characterizes real-time, dynamic, and multisentinel monitoring by combining large data and artificial intelligence.

## Supplementary information

Supplementary information
