# Author Correction: Pretreatment-Integration for Milk Protein Removal and Device-Facilitated Immunochromatographic Assay for 17 Items

**DOI:** 10.1038/s41598-020-78536-3

**Published:** 2020-12-04

**Authors:** Zhiwei Qie, Ziwei Huang, Zichen Gao, Wu Meng, Yanhui Zhu, Rui Xiao, Shengqi Wang

**Affiliations:** Beijing Institute of Radiation Medicine, Beijing, 100850 People’s Republic of China

Correction to: *Scientific Reports* 10.1038/s41598-019-47692-6, published online 12 August 2019

The previous version of this Article contained an incorrect email address for Shengqi Wang, which was incorrectly listed as 87998322@qq.com. This has now been corrected in the HTML and PDF versions of the Article, and in the accompanying Supplementary Information file.

