# Supplementary material for: Pretreatment-Integration for Milk Protein Removal and Device-Facilitated Immunochromatographic Assay for 17 Items
Source: Sci Rep. 2019 Aug 12;9:11630. doi: 10.1038/s41598-019-47692-6 (PMC6690872; doi:10.1038/s41598-019-47692-6)
Supplement: Supplementary file 1 — Supplementary information [file 41598_2019_47692_MOESM1_ESM.docx]

Pretreatment-Integration for milk protein removal and Device-Facilitated Immunochromatographic Assay for 17 items

Zhiwei Qie, Ziwei Huang, Zichen Gao, Wu Meng, Yanhui Zhu, RuiXiao*，Shengqi Wang*

Beijing Institute of Radiation Medicine, Beijing 100850, People`s Republic of China

* Corresponding Authors

Fax: +86-010-66931422. E-mail：ruixiao203@sina.com; Fax: +86-010-66931422. E-mail：[87998322@qq.com](mailto:87998322@qq.com).

The supplementary information is about the calibrations for the other 16 items in milk with and without proposed pretreatment.

**
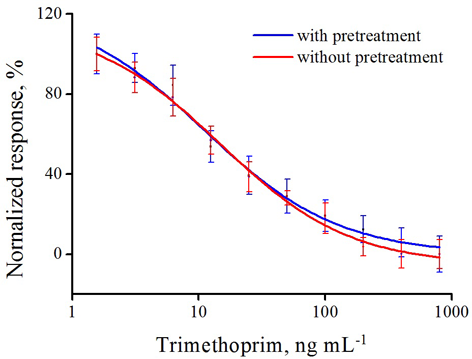
**

**Figure - S1**

**
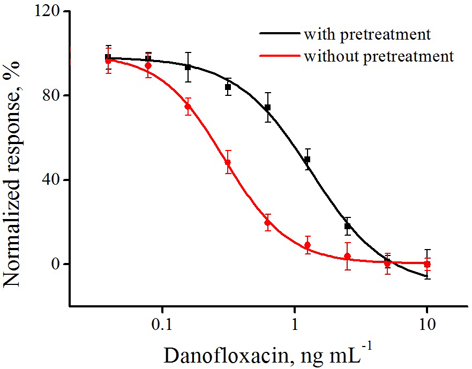
**

**Figure – S2**

**
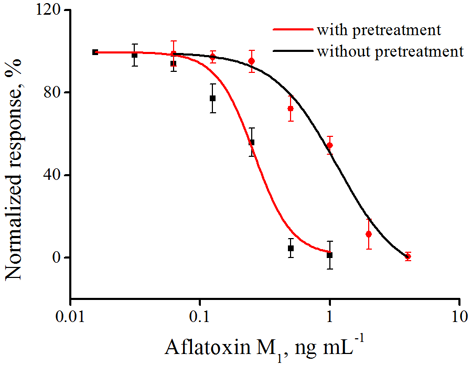
**

**Figure – S3**

**
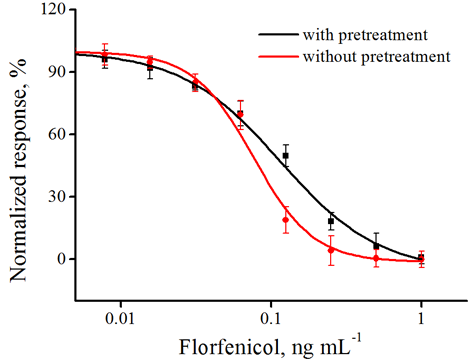
**

**Figure – S4**

**
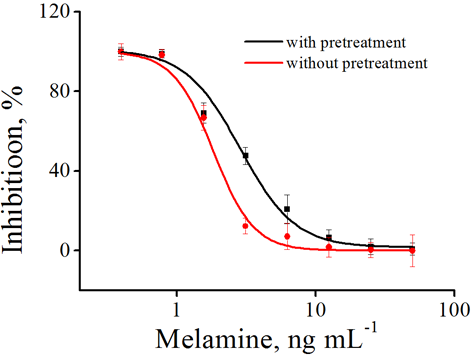
**

**Figure – S5**

**
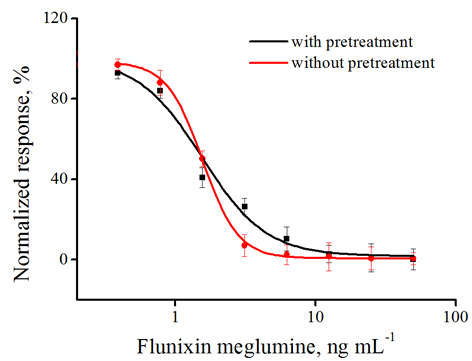
**

**Figure – S6**

**
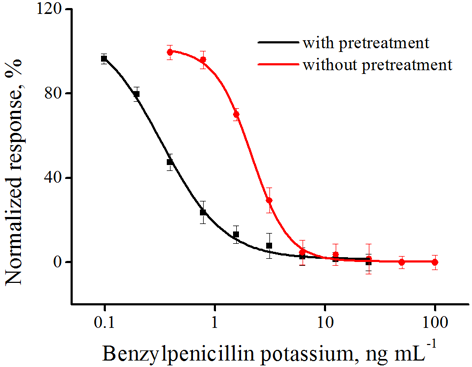
**

**Figure – S7**

**
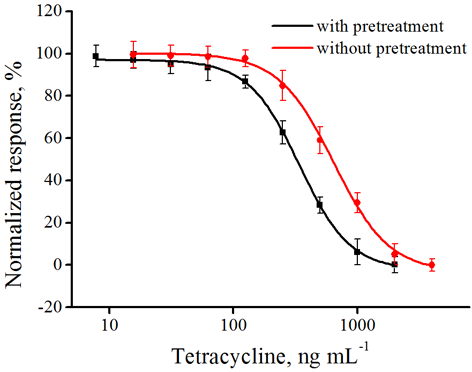
**

**Figure – S8**

**
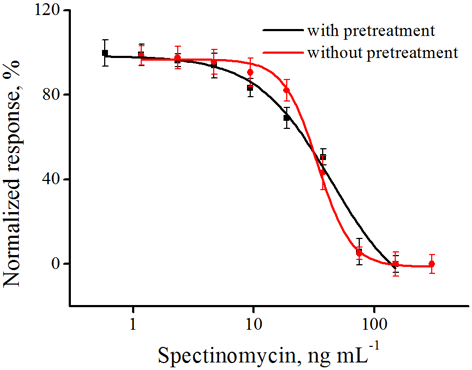
**

**Figure – S9**

**
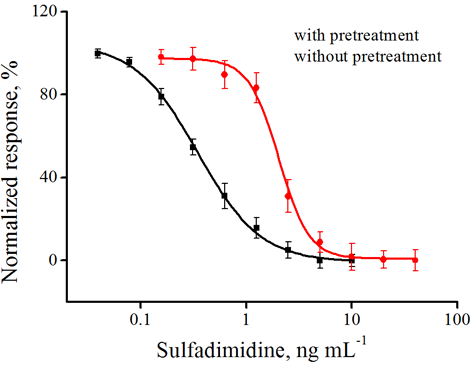
**

**Figure – S10**

**
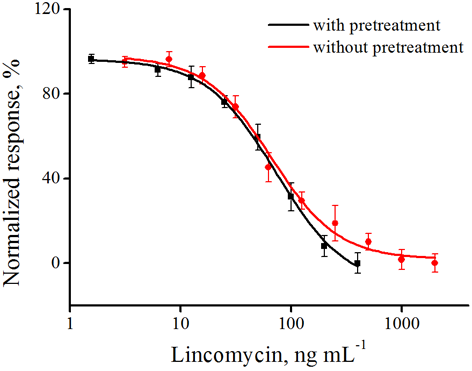
**

**Figure – S11**

**
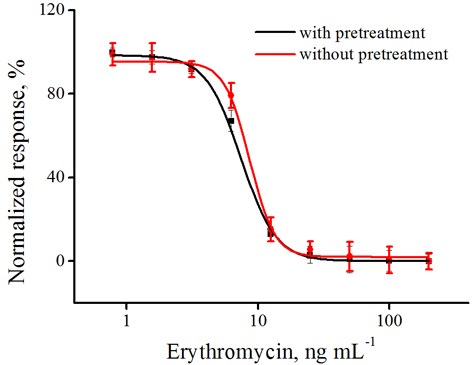
**

**Figure – S12**

**
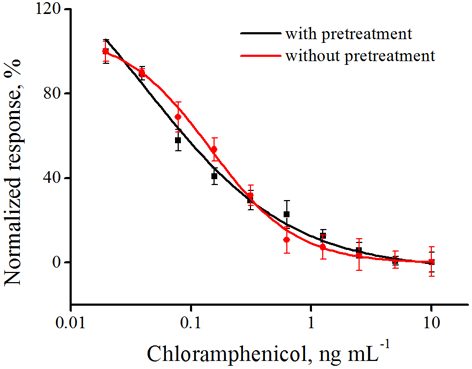
**

**Figure – S13**

**
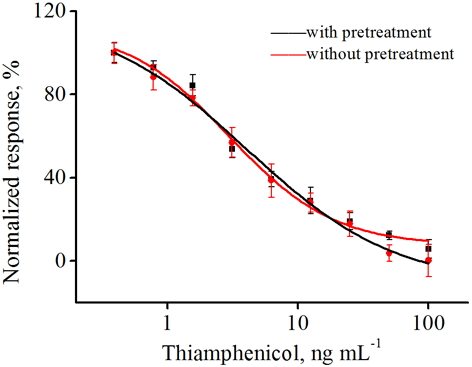
**

**Figure – S14**

**
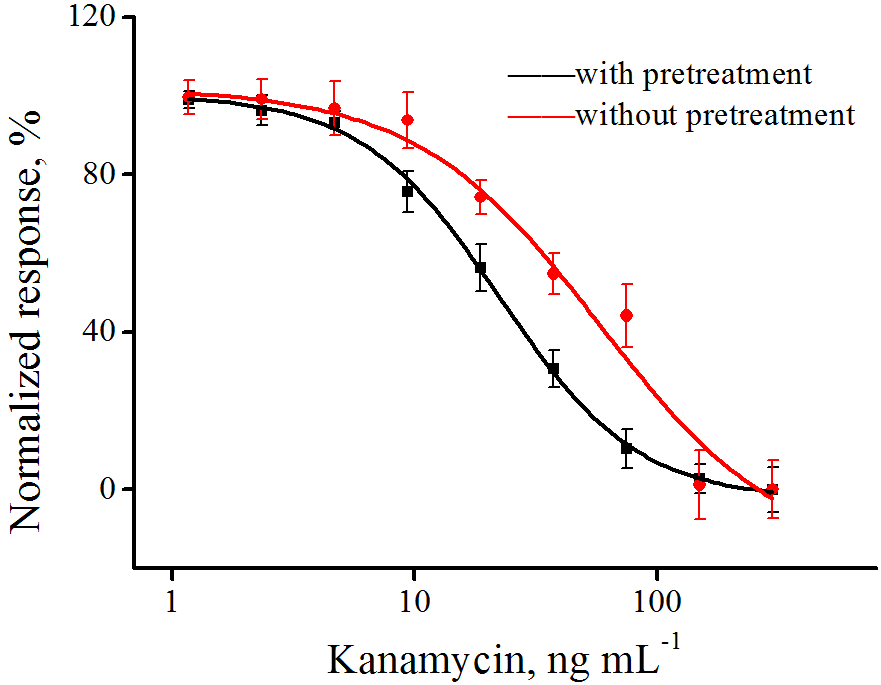
**

**Figure – S15**

**
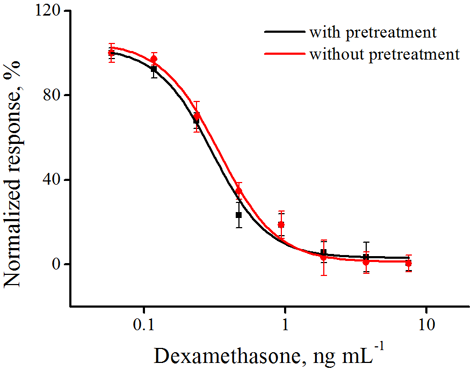
**

**Figure – S16**

**Caption：**

**Figure - S1** The spiked concentrations of trimethoprimwere 0.156, 3.125, 6.25, 12.5, 25, 50, 100, 200, 400, 800 ng mL^-1^

**Figure – S2** The spiked concentrations of danofloxacin were 0.078, 0.156, 0.313, 0.625, 1.25, 2.5, 5, 10 ng mL^-1^

**Figure – S3** The spiked concentrations of aflatoxin M_1_ with pretreatment were 0.0156, 0.0313, 0.0625, 0.125, 0.25, 0.5, 1 ng mL^-1^ and that without pretreatment were 0.0625, 0.125, 0.25, 0.5, 1, 2, 4 ng mL^-1^

**Figure – S4** The spiked concentrations of florfenicol were 1.172, 2.343, 4.688, 9.375, 18.75, 37.5, 75, 150, 300 ng mL^-1^

**Figure – S5** The spiked concentrations of melamine were 0.196, 0.391, 0.781, 1.563, 3.125, 6.25, 12.5, 25, 50 ng mL^-1^

**Figure – S6** The spiked concentrations of flunixin meglumine were 0.781, 1.563, 3.125, 6.25, 12.5, 25, 50, 100, 200 ng mL^-1^

**Figure – S7** The spiked concentrations of benzylpenicillin potassium with pretreatment were 0.195, 0.39, 0.781, 1.563, 3.125, 6.25, 12.5, 25, 50 ng mL^-1^ and that without pretreatment were 0.39, 0.781, 1.563, 3.125, 6.25, 12.5, 25, 50, 100 ng mL^-1^

**Figure – S8** The spiked concentrations of tetracycline with pretreatment were 7.81, 15.63, 31.25, 62.5, 125, 250, 500, 1000, 2000 ng mL^-1^ and that without pretreatment were 15.63, 31.25, 62.5, 125, 250, 500, 1000, 2000, 4000 ng mL^-1^

**Figure – S9** The spiked concentrations of spectinomycin with pretreatment were 0.59, 1.18, 2.35, 4.69, 9.38, 18.75, 37.5, 75, 150 ng mL^-1^ and that without pretreatment were 1.18, 2.35, 4.69, 9.38, 18.75, 37.5, 75, 150, 300 ng mL^-1^

**Figure – S10** The spiked concentrations of sulfadimidine with pretreatment were 0.039, 0.078, 0.156, 0.313, 0.625, 1.25, 2.5, 5, 10 ng mL^-1^ and that without pretreatment were 0.156, 0.313, 0.625, 1.25, 2.5, 5, 10, 20, 40 ng mL^-1^

**Figure – S11** The spiked concentrations of lincomycin with pretreatment were 0.781, 1.563, 3.125, 6.25, 12.5, 25, 50, 100, 200, 400 ng mL^-1^ and that without pretreatment were 3.91, 7.81, 15.63, 31.25, 62.5, 125, 250, 500, 1000, 2000 ng mL^-1^

**Figure – S12** The spiked concentrations of erythromycin were 0.39, 0.781, 1.563, 3.125, 6.25, 12.5, 25, 50, 100, 200 ng mL^-1^

**Figure – S13** The spiked concentrations of chloramphenicol were 0.0195, 0.039, 0.078, 0.156, 0.313, 0.625, 1.25, 2.5, 5, 10 ng mL^-1^

**Figure – S14** The spiked concentrations of thiamphenicol were 0.39, 0.781, 1.563, 3.125, 6.25, 12.5, 25, 50, 100 ng mL^-1^

**Figure – S15** The spiked concentrations of kanamycin were 1.18, 2.35, 4.69, 9.38, 18.75, 37.5, 75, 150, 300 ng mL^-1^

**Figure – S16** The spiked concentrations of dexamethasone were 0.118, 0.235, 0.469, 0.938, 1.88, 3.75, 7.5, 15 ng mL^-1^
